# Clinical Characteristics and Transmission of COVID-19 in Children and Youths During 3 Waves of Outbreaks in Hong Kong

**DOI:** 10.1001/jamanetworkopen.2021.8824

**Published:** 2021-05-03

**Authors:** Gilbert T. Chua, Joshua Sung Chih Wong, Ivan Lam, Polly Po Ki Ho, Wai Hung Chan, Felix Yat Sun Yau, Jaime S. Rosa Duque, Alvin Chi Chung Ho, Ka Ka Siu, Tammy W.Y. Cheung, David Shu Yan Lam, Victor Chi Man Chan, Kwok Piu Lee, Kwing Wan Tsui, Tak Wai Wong, Man Mut Yau, Tsz Yan Yau, Kate Ching Ching Chan, Michelle Wai Ling Yu, Chit Kwong Chow, Wah Keung Chiu, Kwok Chiu Chan, Wilfred H.S. Wong, Marco Hok Kung Ho, Winnie W.Y. Tso, Keith T.S. Tung, Christina S. Wong, Janette Kwok, Wing Hang Leung, Jason C. Yam, Ian C.K. Wong, Paul Kwong Hang Tam, Godfrey Chi Fung Chan, Chun Bong Chow, Kelvin K. W. To, Yu Lung Lau, Kwok Yung Yuen, Patrick Ip, Mike Yat Wah Kwan

**Affiliations:** 1 Department of Paediatrics and Adolescent Medicine, Li Ka Shing Faculty of Medicine, University of Hong Kong, Hong Kong Special Administrative Region, China; 2 Paediatric Infectious Disease Unit, Department of Paediatrics and Adolescent Medicine, Princess Margaret Hospital, Hong Kong Special Administrative Region, China; 3 Department of Paediatrics and Adolescent Medicine, Queen Elizabeth Hospital, Hong Kong Special Administrative Region, China; 4 Department of Paediatrics and Adolescent Medicine, Queen Mary Hospital, Hong Kong Special Administrative Region, China; 5 Department of Paediatrics and Adolescent Medicine, Tuen Mun Hospital, Hong Kong Special Administrative Region, China; 6 Department of Paediatrics and Adolescent Medicine, Pamela Youde Nethersole Eastern Hospital, Hong Kong Special Administrative Region, China; 7 Department of Paediatrics and Adolescent Medicine, Alice Ho Miu Ling Nethersole Hospital, Hong Kong Special Administrative Region, China; 8 Department of Paediatrics and Adolescent Medicine, Tseung Kwan O Hospital, Hong Kong Special Administrative Region, China; 9 Department of Paediatrics, Prince of Wales Hospital, Hong Kong Special Administrative Region, China; 10 Department of Paediatrics and Adolescent Medicine, United Christian Hospital, Hong Kong Special Administrative Region, China; 11 Division of Dermatology, Department of Medicine, Queen Mary Hospital, Hong Kong Special Administrative Region, China; 12 Division of Transplantation and Immunogenetics, Department of Pathology, Queen Mary Hospital, Hong Kong Special Administrative Region, China; 13 Department of Ophthalmology and Visual Sciences, Chinese University of Hong Kong, Hong Kong Special Administrative Region, China; 14 Centre for Safe Medication Practice and Research, Department of Pharmacology and Pharmacy, University of Hong Kong, Hong Kong Special Administrative Region, China; 15 Research Department of Practice and Policy, UCL School of Pharmacy, University College, London, United Kingdom; 16 Division of Paediatric Surgery, Department of Surgery, University of Hong Kong, Hong Kong Special Administrative Region, China; 17 Dr Li Dak-Sum Research Centre, University of Hong Kong-Karolinska, Institutet Collaboration in Regenerative Medicine, University of Hong Kong, Hong Kong Special Administrative Region, China; 18 Department of Microbiology, Carol Yu Centre for Infection, Li Ka Shing Faculty of Medicine, University of Hong Kong, Hong Kong Special Administrative Region, China

## Abstract

**Importance:**

Schools were closed intermittently across Hong Kong to control the COVID-19 outbreak, which led to significant physical and psychosocial problems among children and youths.

**Objective:**

To compare the clinical characteristics and sources of infection among children and youths with COVID-19 during the 3 waves of outbreaks in Hong Kong in 2020.

**Design, Setting, and Participants:**

This cross-sectional study involved children and youths aged 18 years or younger with COVID-19 in the 3 waves of outbreaks from January 23 through December 2, 2020. Data were analyzed from December 2020 through January 2021.

**Main Outcomes and Measures:**

Demographic characteristics, travel and contact histories, lengths of hospital stay, and symptoms were captured through the central electronic database. Individuals who were infected without recent international travel were defined as having domestic infections.

**Results:**

Among 397 children and youths confirmed with COVID-19 infections, the mean (SD) age was 9.95 (5.34) years, 220 individuals (55.4%) were male, and 154 individuals (38.8%) were asymptomatic. There were significantly more individuals who were infected without symptoms in the second wave (59 of 118 individuals [50.0%]) and third wave (94 of 265 individuals [35.5%]) than in the first wave (1 of 14 individuals [7.1%]) (*P* = .001). Significantly fewer individuals who were infected in the second and third waves, compared with the first wave, had fever (first wave: 10 individuals [71.4%]; second wave: 22 individuals [18.5%]; third wave: 98 individuals [37.0%]; *P* < .001) or cough (first wave: 6 individuals [42.9%]; second wave: 15 individuals [12.7%]; third wave: 52 individuals [19.6%]; *P* = .02). Among all individuals, 394 individuals (99.2%) had mild illness. One patient developed chilblains (ie, COVID toes), 1 patient developed multisystem inflammatory syndrome in children, and 1 patient developed post–COVID-19 autoimmune hemolytic anemia. In all 3 waves, 204 patients with COVID-19 (51.4%) had domestic infections. Among these individuals, 186 (91.2%) reported having a contact history with another individual with COVID-19, of which most (183 individuals [90.0%]) were family members. In the third wave, 18 individuals with domestic infections had unknown contact histories. Three schoolmates were confirmed with COVID-19 on the same day and were reported to be close contacts.

**Conclusions and Relevance:**

This cross-sectional study found that nearly all children and youths with COVID-19 in Hong Kong had mild illness. These findings suggest that household transmission was the main source of infection for children and youths with domestic infections and that the risk of being infected at school was small.

## Key Points

**Question:**

What were the major sources of infection among children and youths with COVID-19 in Hong Kong in 2020?

**Findings:**

In this cross-sectional study of 397 children and youths with COVID-19 in the first 3 waves of outbreaks in Hong Kong, in 2020, the largest group had no recent international travel, and nearly all individuals were reported to have other family members with COVID-19. Three students studying in the same school contracted COVID-19, and few children or youths with no recent international travel reported unknown contact histories.

**Meaning:**

These findings suggest that households and not schools were the major route of transmission among children and youths with COVID-19 in Hong Kong.

## Teaser

This cross-sectional study investigates the clinical characteristics and sources of infection of COVID-19 among children and youths in Hong Kong.

## Introduction

In the COVID-19 outbreak, caused by SARS-CoV-2, more than 111 million people have been infected and more than 2.4 million individuals have died worldwide.^1^ The first COVID-19 patient in Hong Kong was diagnosed on January 23, 2020.^2^ Hong Kong is now experiencing its fourth wave of COVID-19 outbreaks, during which multiple public health policies have been implemented to facilitate social distancing to reduce the spread of COVID-19.^3^ These public health policies have had a far greater impact than expected, and nearly all children and youths with COVID-19 in Hong Kong have had only mild illness.^4,5^ During the 4 COVID-19 outbreaks, the Government of Hong Kong implemented territory-wide school closures intermittently.^6,7^ During each school closure, face-to-face teaching was replaced by homeschooling and online classes. Students attended classroom lessons for less than 3 months in 2020. A 2020 large-scale local study^8^ found that the prolonged school closures may have been associated with increased risk among children of developing psychosocial problems, which were associated with decreased emotional and social functioning and decreased physical activity levels. Existing inequalities, such as having children with special educational needs, in families with increased risk of psychosocial problems were associated with increased levels of these adverse outcomes. A 2020 study^9^ in the US found that SARS-CoV-2 infection in children and youths was not associated with attending school or childcare centers, and a 2021 study^10^ in the US found that secondary transmission of SARS-CoV-2 within schools was rare. However, evidence concerning the COVID-19 outbreaks in schools in Hong Kong is lacking. Furthermore, To et al^11^ found that the predominant circulating strains of SARS-CoV-2 in Hong Kong were different in each of the 3 waves, which could possibly influence the clinical characteristics of children and youths infected in the 3 waves. Therefore, we conducted this study to compare the clinical characteristics and identify the sources of infection among children and youths with COVID-19 during the first 3 waves of outbreaks in Hong Kong.

## Methods

The ethics committees of all 7 Hong Kong Hospital Authority clusters approved this cross-sectional study and waived individual informed consent given that the data were collected retrospectively and anonymously. This study followed the Strengthening the Reporting of Observational Studies in Epidemiology (STROBE) reporting guideline.

The study included children and youths aged 18 years or younger with COVID-19 confirmed by the positive detection of SARS-CoV-2 in respiratory specimens by reverse transcriptase polymerase chain reaction (RT-PCR) from January 23 through December 2, 2020. Surveillance criteria during the 3 waves of outbreaks were identical. These children and youths received SARS-CoV-2 RT-PCR testing because they were symptomatic, were close contacts of an individual with a confirmed infection, or were returned international travelers receiving screening at the border. All children and youths with confirmed COVID-19, including those without symptoms, were admitted into the pediatric isolation wards of public hospitals in Hong Kong as part of the mandatory territory-wide infection-control policy.

The physicians (I.L., P.P.K.H., W.H.C., F.Y.S.Y., J.S.R.D., A.C.C.H., K.K.S., T.W.Y.C., D.S.Y.L., V.C.M.C., K.P.L., K.W.T., T.W.W., M.M.Y., T.Y.Y., K.C.C.C., M.W.L.Y., C.K.C., and W.K.C.) in charge of the 9 pediatric isolation wards prospectively collected clinical information for patients upon their discharge as part of the territory-wide pediatric COVID-19 study. The deidentified information was submitted to a centralized electronic data-collection system for analysis. The collected data included sex, age at diagnosis, clinical symptoms, dates of admission and discharge, travel history prior to diagnosis of COVID-19 infection, identifiable contacts with individuals with confirmed COVID-19 infection, and COVID-19–related complications after initial discharge.

### Definitions

We defined the periods of the 3 waves of outbreaks as follows: the first wave was from January 23 through March 21, 2020; the second wave was from March through July 4, 2020; and the third wave was from July 5 through December 2, 2020. All individuals were classified as having domestic infections (ie, with no recent international travel history) or imported infections (ie, with recent travel history outside of the Hong Kong Special Administrative Region and Mainland China within 2 weeks prior to symptom onset). The hospital length of stay was defined as the period from admission to discharge. Patients discharged before July 6, 2020, were required to have 2 negative nasopharyngeal swab SARS-CoV-2 RT-PCR tests taken at least 24 hours apart before being discharged from the hospital. Patients testing positive had repeat RT-PCR tests every 2 to 3 days until the nasopharyngeal swab results were negative. The discharge criterion was modified on July 6, 2020, to any detectable SARS-CoV-2 antinucleoprotein immunoglobulin G (IgG) antibodies in the serum regardless of a positive respiratory specimen RT-PCR result.

### Statistical Analysis

Statistical analyses were performed using SPSS statistical software version 26 (IBM) from December 2020 through January 2021. We used χ^2^ exact test to compare differences in clinical symptoms. One-way analysis of variances was used to detect differences in the mean age at diagnosis and mean length of hospital stay among patients in the 3 waves of outbreaks. The hospital admissions during the study period were presented as plots by admission dates. A 2-tailed *P* value less than .05 was considered statistically significant.

## Results

From January 23 through December 2, 2020, a total of 397 children and youths with COVID-19 were captured in the central database and were included in this study; 220 individuals (55.4%) were male, the mean (SD) age was 9.95 (5.34) years, and 154 individuals (38.8%) were asymptomatic. In all 3 waves, 204 patients with COVID-19 (51.4%) had domestic infections. Among these individuals, 186 (91.2%) reported having a contact history with another individual with COVID-19, of which most (183 individuals [90.0%]) were family members. The Figure shows the number of children and youths with COVID-19 admitted during the 3 waves of COVID-19 outbreaks in association with the school closure periods: 14 individuals were confirmed in the first wave, 118 individuals in the second wave, and 265 individuals in the third wave. Among individuals in the first and second waves, most were classified as having imported infections (first wave: 11 individuals [78.6%]; second wave: 110 individuals [93.2%]), whereas most individuals in the third wave were classified with domestic infections (193 individuals [72.8%]).

**Figure.**
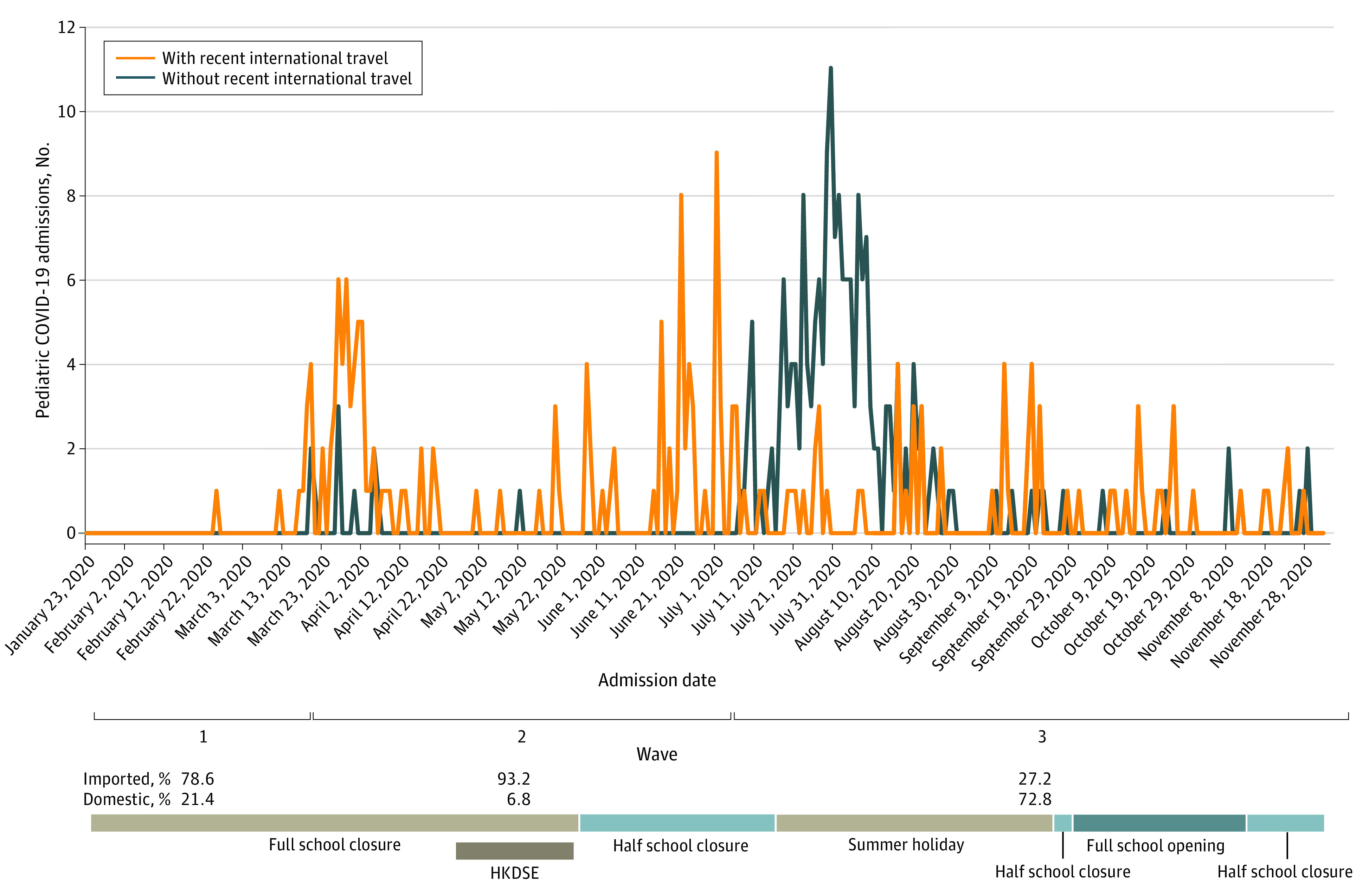
Admissions of Children and Youths With COVID-19 in Hong Kong Kindergarten year 1 and 2 students were not allowed to return to school from January 24 through September 29, 2020. Secondary year 3 to 5 students resumed classes on May 27, 2020; primary year 4 to 6 and secondary year 1 and 2 students resumed classes on June 8, 2020; kindergarten year 3 and primary year 1 to 3 students resumed classes on June 15, 2020. Summer holiday began on July 14, 2020, and extended through September 23, 2020. Kindergarten year 3; primary year 1, 5, and 6; and secondary year 1, 5, and 6 students resumed classes on September 23, 2020; kindergarten year 1 and 2, primary year 2 to 4, and secondary year 2 to 4 students resumed classes on September 29, 2020. Kindergarten year 1 to 3 and primary year 1 to 3 classes were closed on November 14 and November 23, 2020, respectively, owing to outbreaks of non-COVID-19–related upper respiratory tract infections; primary year 4 to 6 and all secondary school classes were closed on December 2, 2020, owing to the wave 4 COVID-19 outbreak. HKDSE indicates Hong Kong Diploma of Secondary Education examination period.

Table 1 shows the clinical characteristics of children and youths with COVID-19 in the 3 waves of outbreaks. Patients diagnosed in the third wave were significantly younger by mean (SD) age (9.0 [5.1] years) than those in the first wave (11.6 [6.4] years) and second wave (11.8 [5.2] years) (*P* < .001). In the first and second waves, most ethnic Chinese Hong Kong residents with international travel were returning from the United Kingdom (8 individuals [72.7%] in the first wave and 50 individuals [45.5%] in the second wave). In the third wave, 72 individuals (27.2%) had recent international travel, with most ethnic Indian Hong Kong residents with international travel returning from India (48 individuals [66.7%]). There were significantly more individuals without symptoms in the second wave (59 individuals [50.0%]; odds ratio [OR], 13.0; 95% CI, 1.6-102.6) and third wave (94 individuals [35.5%]; OR, 7.1; 95% CI, 0.92-55.4) compared with the first wave (1 individual [7.1%]) (*P* = .001). Fewer than 10% of patients presented with anosmia over all 3 waves (17 individuals [4.3%]) or ageusia (14 individuals [3.5%]). Patients treated in the third wave had significantly shorter mean (SD) hospital lengths of stay (9.0 [5.1] days) compared with individuals in the first wave (24.0 [13.3] days) and second wave (18.1 [9.6] days) (*P* < .001). This difference was likely associated with the change in discharge criteria from 2 consecutive negative results for nasopharyngeal swab SARS-CoV-2 RT-PCR to the detection of serum antinucleoprotein IgG antibodies. Significantly fewer individuals who were infected in the second and third waves, compared with the first wave, had fever (first wave: 10 individuals [71.4%]; second wave: 22 individuals [18.5%]; third wave: 98 individuals [37.0%]; P < .001) or cough (first wave: 6 individuals [42.9%]; second wave: 15 individuals [12.7%]; third wave: 52 individuals [19.6%]; *P* = .02).

**Table 1.** Characteristics and Travel History of Children and Youths With COVID-19

Characteristic	Individuals infected, No. (%)			*P* value
	First wave^a^ (n = 14)	Second wave^b^ (n = 118)	Third wave^c^ (n = 265)	
Age, mean (SD), y	11.6 (6.4)	11.8 (5.2)	9.0 (5.1)	<.001
Sex				
Male	7 (50.0)	70 (59.3)	143 (54.0)	.57
Female	7 (50.0)	48 (40.7)	122 (46.0)	
With international travel	11 (78.6)	110 (93.2)	72 (27.2)	<.001
Mainland China	1 (9.1)	0	0	<.001
Japan	1 (9.1)	0	0	<.001
India	0	16 (14.5)	48 (66.7)	<.001
Other parts of Asia^d^	1 (9.1)	40 (36.4)	19 (26.4)	.10
United Kingdom	8 (72.7)	50 (45.5)	2 (2.8)	<.001
Switzerland	1 (9.1)	0	0	<.001
Spain	0	1 (0.9)	0	.68
Other parts of Europe^e^	1 (9.1)	4 (3.6)	3 (4.2)	.69
Canada	0	1 (0.9)	0	.68
South America	0	0	1 (1.4)	.43
United States of America	0	4 (3.6)	0	.21
Hospital length of stay, mean (SD), d	24.0 (13.3)	18.1 (9.6)	9.0 (5.1)	<.001
Symptoms				
Asymptomatic	1 (7.1)	59 (50.0)	94 (35.5)	.001
Fever	10 (71.4)	22 (18.6)	98 (37.0)	<.001
Cough	6 (42.9)	15 (12.7)	52 (19.6)	.02
Sputum	4 (28.6)	5 (4.2)	11 (4.2)	<.001
Rhinorrhea	3 (21.4)	15 (12.7)	46 (17.4)	.45
Sneezing	1 (7.1)	4 (3.4)	7 (2.6)	.61
Blocked nose	2 (14.3)	4 (3.4)	7 (2.6)	.07
Ageusia	1 (7.1)	5 (4.2)	8 (3.0)	.63
Anosmia	1 (7.1)	6 (5.1)	10 (3.8)	.73
Loss of appetite	3 (21.4)	2 (1.7)	5 (1.9)	<.001
Vomiting	0	1 (0.8)	13 (4.9)	.11
Diarrhea	2 (14.3)	9 (7.6)	16 (6.0)	.45
Rash	0	1 (0.8)	0	.31

^a^ January 23 to March 21, 2020.

^b^ March 22 to July 4, 2020.

^c^ July 5 to December 2. 2020.

^d^ Includes Singapore, Pakistan, Dhaka, Kazakhstan, Russia, Nepal, the Philippines, and Dubai.

^e^ Includes Sweden, Scotland, the Netherlands, Ukraine, Ireland, and the Czech Republic.

None of our patients developed pneumonia or required oxygen or intensive care treatment. Among all individuals, 394 individuals (99.2%) had mild illness. All patients recovered without complications, except 3 patients in the first wave who presented with unusual manifestations associated with COVID-19. One of these patients developed multiple chilblains on the toes, which were resolved in 1 week with conservative management. The second patient, an ethnic non-Chinese individual, developed multisystem inflammatory syndrome in children (MIS-C) approximately 4 weeks after being diagnosed with the infection. He presented with fever, sore throat, cervical lymphadenopathy, bilateral conjunctival injection, cracked lips, and strawberry tongue but without cardiac complications. He was treated successfully with 2 doses of intravenous immunoglobulin. The third patient in the first wave developed steroid-dependent autoimmune hemolytic anemia 3 months after being diagnosed with the infection and achieved remission with 4 doses of rituximab.

Table 2 shows the contact histories of the individuals with domestic and imported COVID-19 infections. Most patients overall (273 individuals [68.8%]) reported having a contact history with another individual confirmed to have COVID-19, with most of those having infected family members (270 individuals [98.9%]), mainly parents, grandparents, and siblings. In the first wave, 3 individuals with imported infections reported having contacts with other infected schoolmates at overseas boarding schools, as did 6 individuals in the second wave. In the third wave, 3 individuals confirmed with domestic infections on the same day reported having contacts with schoolmates confirmed to have COVID-19; these contacts occurred more than 1 month after the May 27, 2020, school reopening. The first patient, whose parents and their domestic helper were also confirmed to have COVID-19, had contact with the second patient, playing volleyball and having a meal together without wearing a mask. They developed symptoms 5 days apart. The third patient developed symptoms the same day as the second patient. She was initially unaware of any COVID-19 contacts but was subsequently identified by the medical team to be a student at the same school and in the same grade as the first 2 patients. Among individuals with imported infections, from 4 individuals in the first wave (36.4%) to 41 individuals in the third wave (56.9%) were uncertain about their contact histories, whereas 18 individuals with domestic infections in the third wave (9.3%) reported no known contact history. Significantly more individuals presenting with diarrhea reported having contacts with more than 2 individuals confirmed with COVID-19 compared with individuals without diarrhea (12 of 27 individuals [44.4%] vs 54 of 370 [14.6%] individuals; *P* < .001).

**Table 2.** Contact Histories of Children and Youths With COVID-19

	Individuals infected, No. (%)			*P* value
	First wave^a^	Second wave^b^	Third wave^c^	
**Without international travel (n = 204)**				
Total	3	8	193	NA
Father	1 (33.3)	4 (50.0)	93 (48.2)	.87
Mother	2 (66.7)	7 (87.5)	100 (51.8)	.13
Sibling	0	5 (62.5)	64 (33.2)	.11
Grandparent	0	1 (12.5)	52 (26.9)	.39
Cousin	0	0	5 (2.6)	.86
Uncle or aunt	1 (33.3)	0	15 (7.8)	.19
Schoolmate	0	0	3 (1.0)	.92
Friend	0	0	2 (1.0)	.94
Unknown	0	0	18 (9.3)	.57
**With international travel (n = 193)**				
Total	11	110	72	NA
Father	4 (36.4)	12 (10.9)	14 (19.4)	.04
Mother	2 (18.2)	24 (21.8)	20 (27.8)	.59
Sibling	1 (9.1)	21 (19.1)	16 (22.2)	.58
Grandparent	3 (27.3)	3 (2.7)	4 (5.6)	.002
Cousin	0	1 (0.9)	1 (1.4)	.90
Uncle or aunt	0	3 (2.7)	4 (5.6)	.49
Schoolmate	3 (27.3)	6 (5.5)	0	<.001
Friend	0	5 (4.5)	0	.14
Unknown	4 (36.4)	61 (55.5)	41 (56.9)	.44

Abbreviation: NA, not applicable.

^a^ January 23 to March 21, 2020.

^b^ March 22 to July 4, 2020.

^c^ July 5 to December 2, 2020.

## Discussion

To our knowledge, this cross-sectional study is the first study comparing the clinical characteristics and transmission patterns among children and youths with COVID-19 in East Asia in 2020. We found significant differences in the clinical presentations across the 3 waves of outbreaks. There were more individuals who were infected without symptoms in the second and third waves than in the first wave. Fewer patients diagnosed in the second and third waves had symptoms (ie, fever and cough) than patients in the first wave. The screening criteria for individuals without symptoms who were carriers of SARS-CoV-2 and close contacts of individuals infected with COVID-19 were the same in the 3 waves of outbreaks; therefore, the differences in the percentages of individuals without symptoms who were carriers in the 3 waves were unlikely to be associated with a change in public health policies. On the contrary, this might be associated with the different predominant SARS-CoV-2 strains circulating in the 3 waves. The study by To et al^11^ looked at the viral genome of the SARS-CoV-2 strains in the 3 waves of outbreaks in Hong Kong. The spike protein D614G mutation, which is associated with increased infectivity but not increased disease severity,^12,13^ was not found in patient samples during the first wave, but it was present in 73.8% of samples in the second wave. It was predominantly found in travelers returning from North America and Europe. The circulating strains in 87.9% of the individuals with domestic infections in the third wave belonged to the Global Initiative on Sharing All Influenza Data clade GR, Nextstrain clade 20B, and Pangolin lineage B.1.1, in addition to D614G.^11^ Children and youths presenting with diarrhea were more likely to report having contact with more than 2 infected individuals, which could be associated with the exposure to a higher viral load, given that a previous study found a higher viral load in the stools of patients with diarrhea^14^

This study also identified 3 patients with rare manifestations of COVID-19. Chilblains, also known as COVID toes, presenting in both children and adults is a dermatological manifestation recognized as a symptom of COVID-19.^15^ Although the exact pathophysiology of chilblains in individuals with COVID-19 remains to be elucidated, possible mechanisms include an abnormal inflammatory response attributed to type I interferonopathies or thrombotic microvasculature induced by complement activation and procoagulation state.^16^ Another unique manifestation of COVID-19 was MIS-C, with features resembling those of Kawasaki disease, which developed 4 weeks after diagnosis of the infection in a patient who was not Chinese. However, no other Hong Kong Chinese individuals with COVID-19 developed MIS-C. Globally, the first case of MIS-C as a complication of pediatric COVID-19 was reported in Europe.^17^ Multiple pediatric studies in Asia have reported that MIS-C is extremely rare, if not absent, among East Asian populations, despite Kawasaki disease being significantly more prevalent in Asia than in the rest of the world.^4,5,18^ Multinational collaborative studies are needed to explain the genetic predisposition for MIS-C across different ethnicities. It is recommended that pediatricians in Asia treating children and youths who have COVID-19 and are not East Asian consider the development of MIS-C after the primary infection. We also reported a patient with COVID-19 who developed steroid-dependent autoimmune hemolytic anemia 3 months after diagnosis of the infection and achieved remission with rituximab. The study team is conducting an ongoing investigation to look at possible genetic causes for this patient. Post-COVID-19 autoimmune hemolytic anemia has been documented in several adult case report series^19^ and was reported in a teenager with symptoms suggestive of autoimmune lymphoproliferative syndrome.^20^ Pediatricians should follow up with patients and provide adequate counseling to parents with regard to long-term complications that might develop after the primary infection with COVID-19.

Our study found that transmission of SARS-CoV-2 within school campuses in Hong Kong was rare, regardless of whether the schools were closed or reopened. Only 3 individuals with domestic COVID-19 infections in our study were reported to be schoolmates in close contact. There were no reports that teachers had transmitted COVID-19 to their students. Notably, most of the pediatric patients had imported infections in the first and second waves. They were students returning to Hong Kong from overseas boarding schools who had contact with infected schoolmates. After the closure of schools, many students switched their learning to online platforms, with significant physical and social outcomes. A recent study conducted by the Hong Kong Eye Hospital found that myopia progression in children during the home confinement period was significantly greater than in the pre-COVID-19 period (X.J. Zhang, PhD, unpublished data, 2021). Tso et al^8^ found that school closures in Hong Kong were associated with significant changes in the physical and psychosocial well-being of children, and the differences in outcomes were increased among families of children with special educational needs, families with members who had mental health problems, single-parent families, and families with lower incomes. School closures at critical periods in the educational development of primary school children have also been associated with deleterious outcomes, such as decreased educational attainment and life expectancies.^21,22^ The Hong Kong Diploma of Secondary Education (HKDSE) examination held from April 24 through May 25, 2020, was attended by more than 50 000 secondary school students in person. Candidates were not allowed to attend if they were undergoing mandatory quarantine. Examination venues were disinfected regularly and had adequate ventilation. Candidates’ temperatures were measured before they could enter the examination hall, and they were required to maintain social distancing of at least 1.8 m and wear masks during the examination. There were no reports of students being infected during the examinations.^23^ In addition, the sources of infection for most individuals in our study group were family members and not schools, and it is typical in Hong Kong that extended family members reside in the same household.

A peak in domestic infections between the middle of July and August 2020 led to a third wave of domestic outbreak. It was primarily attributed to the exemption of several groups of returning travelers, including seafarers and aircrew, from COVID-19 screening and quarantine. These unscreened returning travelers spread the virus to local adults within the community and became the sources of infection for children and youths at home while schools were closed.^24^ Instead of closing schools, which could lead to multiple short-term and long-term detrimental outcomes, public health policies should focus on preventing adults in the community from becoming infected and transmitting COVID-19 to children and youths. This could be facilitated by public health measures implemented in Hong Kong, such as universal mask wearing, which have not yet been widely implemented in Europe or North America.^25^ Furthermore, Wang et al^26^ found that household transmission of SARS-CoV-2 was 18-fold higher with frequent close contact with an infected person. Other effective public health measures, including travel restrictions, border control, and social distancing, should be enforced to prevent the importation of infections and transmission within the community.^6,7,27,28^ Furthermore, when the transmission rate is low, schools could be reopened cautiously^29^ and in a staged manner, with strict physical distancing within the campus and regular disinfection with chlorine or alcohol.^26^ Additional measures should also be implemented in schools, such as installing plastic partitions between classroom desks or canteen tables, minimizing group activities, reinforcing infection-control measures like teaching students good hand hygiene and monitoring symptoms, providing basic protective equipment to teachers, and incorporating digital teaching tools in classrooms.^30^ Activities that may require removal of face masks, such as vigorous exercise and sports that involve close body contact, should be suspended. Schools could consider opening for half days so that students do not need to stay at school for lunch. Alternatively, students could have their lunch at their desks with plastic partitions installed. Schools should also consider asking parents to declare whether other family members have been in recent contact with infected individuals. Students with contact histories should not return to school until they test negative. Students under quarantine at home could be supported by online learning.

### Limitations

The study has several limitations, and data from this study should be interpreted with the following caveats. Although there were 488 patients confirmed with COVID-19 infection by the end of the third wave, some patients in the third wave had not yet been discharged from isolation facilities and were not yet captured in our database. We have yet to include the clinical characteristics of the individuals’ family members who also had COVID-19. Hong Kong is a unique city in the Southeast Asia, with many travelers coming from overseas, as well as tens of thousands of Hong Kong children and youths studying abroad and returning to Hong Kong several times per year. This could make it difficult to generalize our findings to other cities in China and Asia. Additionally, the number of patients in the first wave was small. Nevertheless, we have included all Hong Kong children and youths with COVID-19 in the first and second waves, and the criteria implemented in Hong Kong for the screening of individuals without symptoms who were carriers and of close contacts of individuals who were infected remained the same throughout the 3 waves of outbreak.

## Conclusions

In this cross-sectional study, children and youths with COVID-19 had a wide range of clinical presentations, from no symptoms to postinfectious immune-mediated complications. Recent reports found that a new SARS-CoV-2 strain, the variant of concern 202012/01, has emerged in the United Kingdom, which is likely to be more transmissible than existing circulating strains.^31^ Whether this new circulating strain will lead to a massive increase in global transmissions or to a new clinical spectrum of diseases remains to be investigated. Nevertheless, our study found that nearly all children and youths with COVID-19 were infected through household transmission. Risk of being infected at school was very small if social distancing and face mask policies were enforced. Given that school closures are associated with negative short-term and long-term outcomes among children and their families, school closures as a first-line measure in preventing the spread of SARS-CoV-2 within the community should be considered very carefully and their effectiveness should be reviewed regularly.

## References

[1] WHO coronavirus disease (COVID-19) dashboard. World Health Organization. Accessed February 25, 2021. https://covid19.who.int/

[2] Leung GM, Cowling BJ, Wu JT. From a sprint to a marathon in Hong Kong. N Engl J Med. 2020;382(18):e45. doi:10.1056/NEJMc2009790 32294373 PMC7179992

[3] Cowling BJ, Ali ST, Ng TWY, . Impact assessment of non-pharmaceutical interventions against coronavirus disease 2019 and influenza in Hong Kong: an observational study. Lancet Public Health. 2020;5(5):e279-e288. doi:10.1016/S2468-2667(20)30090-6 32311320 PMC7164922

[4] Xiong X, Chua GT, Chi S, . A comparison between Chinese children infected with coronavirus disease-2019 and with severe acute respiratory syndrome 2003. J Pediatr. 2020;224:30-36. doi:10.1016/j.jpeds.2020.06.041 32565097 PMC7301144

[5] Chua GT, Xiong X, Choi EH, . COVID-19 in children across three Asian cosmopolitan regions. Emerg Microbes Infect. 2020;9(1):2588-2596. doi:10.1080/22221751.2020.1846462 33138739 PMC7723019

[6] Chu DK, Akl EA, Duda S, Solo K, Yaacoub S, Schünemann HJ; COVID-19 Systematic Urgent Review Group Effort (SURGE) study authors. Physical distancing, face masks, and eye protection to prevent person-to-person transmission of SARS-CoV-2 and COVID-19: a systematic review and meta-analysis. Lancet. 2020;395(10242):1973-1987. doi:10.1016/S0140-6736(20)31142-9 32497510 PMC7263814

[7] Wong SYS, Kwok KO, Chan FKL. What can countries learn from Hong Kong’s response to the COVID-19 pandemic? CMAJ. 2020;192(19):E511-E515. doi:10.1503/cmaj.200563 32332040 PMC7234274

[8] Tso WWY, Wong RS, Tung KTS, . Vulnerability and resilience in children during the COVID-19 pandemic. Eur Child Adolesc Psychiatry. 2020;1-16. doi:10.1007/s00787-020-01680-8 33205284 PMC7671186

[9] Hobbs CV, Martin LM, Kim SS, ; CDC COVID-19 Response Team. Factors associated with positive SARS-CoV-2 test results in outpatient health facilities and emergency departments among children and adolescents aged <18 Years—Mississippi, September-November 2020. MMWR Morb Mortal Wkly Rep. 2020;69(50):1925-1929. doi:10.15585/mmwr.mm6950e3 33332298 PMC7745952

[10] Zimmerman KO, Akinboyo IC, Brookhart MA, ; ABC Science Collaborative. Incidence and secondary transmission of SARS-CoV-2 infections in schools. Pediatrics. 2021;e2020048090. doi:10.1542/peds.2020-048090 33419869 PMC8015158

[11] To KK-W, Chan W-M, Ip JD, . Unique clusters of severe acute respiratory syndrome coronavirus 2 causing a large coronavirus disease 2019 outbreak in Hong Kong. Clin Infect Dis. 2020;ciaa1119. doi:10.1093/cid/ciaa1119 32756996 PMC7454385

[12] Korber B, Fischer WM, Gnanakaran S, ; Sheffield COVID-19 Genomics Group. Tracking changes in SARS-CoV-2 spike: evidence that D614G increases infectivity of the COVID-19 virus. Cell. 2020;182(4):812-827.e19. doi:10.1016/j.cell.2020.06.043 32697968 PMC7332439

[13] Plante JA, Liu Y, Liu J, . Spike mutation D614G alters SARS-CoV-2 fitness. Nature. 2020. doi:10.1038/s41586-020-2895-3 33106671 PMC8158177

[14] Cheung KS, Hung IFN, Chan PPY, . Gastrointestinal manifestations of SARS-CoV-2 infection and virus load in fecal samples from a Hong Kong cohort: systematic review and meta-analysis. Gastroenterology. 2020;159(1):81-95. doi:10.1053/j.gastro.2020.03.065 32251668 PMC7194936

[15] Mohan V, Lind R. Chilblains in COVID-19 infection. Cureus. 2020;12(7):e9245-e9245. doi:10.7759/cureus.9245 32821591 PMC7430661

[16] Ladha MA, Luca N, Constantinescu C, Naert K, Ramien ML. Approach to chilblains during the COVID-19 pandemic [formula: see text]. J Cutan Med Surg. 2020;24(5):504-517. doi:10.1177/1203475420937978 32741218

[17] To KKW, Chua GT, Kwok KL, . False-positive SARS-CoV-2 serology in 3 children with Kawasaki disease. Diagn Microbiol Infect Dis. 2020;98(3):115141. doi:10.1016/j.diagmicrobio.2020.115141 32795776 PMC7366972

[18] Han MS, Choi EH, Chang SH, . Clinical characteristics and viral RNA detection in children with coronavirus disease 2019 in the Republic of Korea. JAMA Pediatr. 2021;175(1):73-80. doi:10.1001/jamapediatrics.2020.3988 32857112 PMC7455883

[19] Lazarian G, Quinquenel A, Bellal M, . Autoimmune haemolytic anaemia associated with COVID-19 infection. Br J Haematol. 2020;190(1):29-31. doi:10.1111/bjh.16794 32374906 PMC7267601

[20] Wahlster L, Weichert-Leahey N, Trissal M, Grace RF, Sankaran VG. COVID-19 presenting with autoimmune hemolytic anemia in the setting of underlying immune dysregulation. Pediatr Blood Cancer. 2020;67(9):e28382. doi:10.1002/pbc.28382 32495391 PMC7674227

[21] Christakis DA, Van Cleve W, Zimmerman FJ. Estimation of US children’s educational attainment and years of life lost associated with primary school closures during the coronavirus disease 2019 pandemic. JAMA Netw Open. 2020;3(11):e2028786-e2028786. doi:10.1001/jamanetworkopen.2020.28786 33180132 PMC7662136

[22] Graves JM, Mackelprang JL, Abshire DA. Coronavirus disease 2019 and effects of school closure for children and their families. JAMA Pediatr. 2021;175(2):210-211. doi:10.1001/jamapediatrics.2020.3589 33226421 PMC9242551

[23] Hong Kong Examinations and Assessment Authority. 2020 Hong Kong diploma of secondary education examination (HKDSE) contingency and precautionary measures at examination centres. Updated April 9, 2020. Accessed December 30, 2020. http://www.hkeaa.edu.hk/DocLibrary/MainNews/Instructions_to_Candidates_Precautionary_Measures_at_Exam_Centre_Eng_.pdf

[24] Third wave likely originated from imported cases or quarantine exemptions, HKU expert says. University of Hong Kong. Accessed February 25, 2021. https://fightcovid19.hku.hk/third-wave-likely-originated-from-imported-cases-or-quarantine-exemptions-hku-expert-says/

[25] Klompas M, Morris CA, Sinclair J, Pearson M, Shenoy ES. Universal masking in hospitals in the Covid-19 era. N Engl J Med. 2020;382(21):e63. doi:10.1056/NEJMp2006372 32237672

[26] Wang Y, Tian H, Zhang L, . Reduction of secondary transmission of SARS-CoV-2 in households by face mask use, disinfection and social distancing: a cohort study in Beijing, China. BMJ Glob Health. 2020;5(5):e002794. doi:10.1136/bmjgh-2020-002794 32467353 PMC7264640

[27] Costantino V, Heslop DJ, MacIntyre CR. The effectiveness of full and partial travel bans against COVID-19 spread in Australia for travellers from China during and after the epidemic peak in China. J Travel Med. 2020;27(5):taaa081. doi:10.1093/jtm/taaa081 32453411 PMC7313810

[28] Wells CR, Sah P, Moghadas SM, . Impact of international travel and border control measures on the global spread of the novel 2019 coronavirus outbreak. Proc Natl Acad Sci U S A. 2020;117(13):7504-7509. doi:10.1073/pnas.2002616117 32170017 PMC7132249

[29] Yoon Y, Kim KR, Park H, Kim S, Kim YJ. Stepwise school opening and an impact on the epidemiology of COVID-19 in the children. J Korean Med Sci. 2020;35(46):e414. doi:10.3346/jkms.2020.35.e414 33258334 PMC7707922

[30] Viner RM, Bonell C, Drake L, . Reopening schools during the COVID-19 pandemic: governments must balance the uncertainty and risks of reopening schools against the clear harms associated with prolonged closure. Arch Dis Child. 2021;106(2):111-113. doi:10.1136/archdischild-2020-319963 32747375 PMC7401577

[31] SARS-CoV-2 variant—United Kingdom of Great Britain and Northern Ireland. World Health Organization. Accessed December 22, 2020. https://www.who.int/csr/don/21-december-2020-sars-cov2-variant-united-kingdom/en/

